# Dynamic Function and Composition Changes of Immune Cells During Normal and Pathological Pregnancy at the Maternal-Fetal Interface

**DOI:** 10.3389/fimmu.2019.02317

**Published:** 2019-10-18

**Authors:** Fenglian Yang, Qingliang Zheng, Liping Jin

**Affiliations:** Clinical and Translational Research Center of Shanghai First Maternity and Infant Hospital, Tongji University School of Medicine, Shanghai, China

**Keywords:** maternal-fetus interface, decidua, immune cells, pregnancy, macrophages, NK cells

## Abstract

A successful pregnancy requires a fine-tuned and highly regulated balance between immune activation and embryonic antigen tolerance. Since the fetus is semi-allogeneic, the maternal immune system should exert tolerant to the fetus while maintaining the defense against infection. The maternal-fetal interface consists of different immune cells, such as decidual natural killer (dNK) cells, macrophages, T cells, dendritic cells, B cells, and NKT cells. The interaction between immune cells, decidual stromal cells, and trophoblasts constitute a vast network of cellular connections. A cellular immunological imbalance may lead to adverse pregnancy outcomes, such as recurrent spontaneous abortion, pre-eclampsia, pre-term birth, intrauterine growth restriction, and infection. Dynamic changes in immune cells at the maternal-fetal interface have not been clearly stated. While many studies have described changes in the proportions of immune cells in the normal maternal-fetus interface during early pregnancy, few studies have assessed the immune cell changes in mid and late pregnancy. Research on pathological pregnancy has provided clues about these dynamic changes, but a deeper understanding of these changes is necessary. This review summarizes information from previous studies, which may lay the foundation for the diagnosis of pathological pregnancy and put forward new ideas for future studies.

## Introduction

The decidua also called the maternal-fetus interface (1), is the mucous membrane of the pregnant uterus, and is characterized by immunological tolerance toward the allogeneic fetus and maintenance of host defense against possible pathogens. The decidua originates from differentiated endometrial cells in early pregnancy (2) and encases the fetus, umbilical cord, and placenta. According to the anatomical relationship between decidua and blastocyst, the decidua can be divided into different parts: decidua basalis, parietalis, and capsularis. The former transforms from the endometrium of the embryo implantation site and covers the basal plate of the placenta, the middle lines the fetal membranes; the latter covers other parts of the uterine cavity (3). The maternal-fetus interface consists of decidual stromal cells, decidual immune cells, and trophoblast cells. This interface tolerates the semi-allogeneic fetus as well as retain the ability to defend a pathogen infection locally (4, 5). It plays a crucial role in mediating O_2_, CO_2_, and nutrients (6), exerting immunological protection; and generating different hormones, enzymes, and cytokines, to establish a successful pregnancy (7).

**Figure 1:**
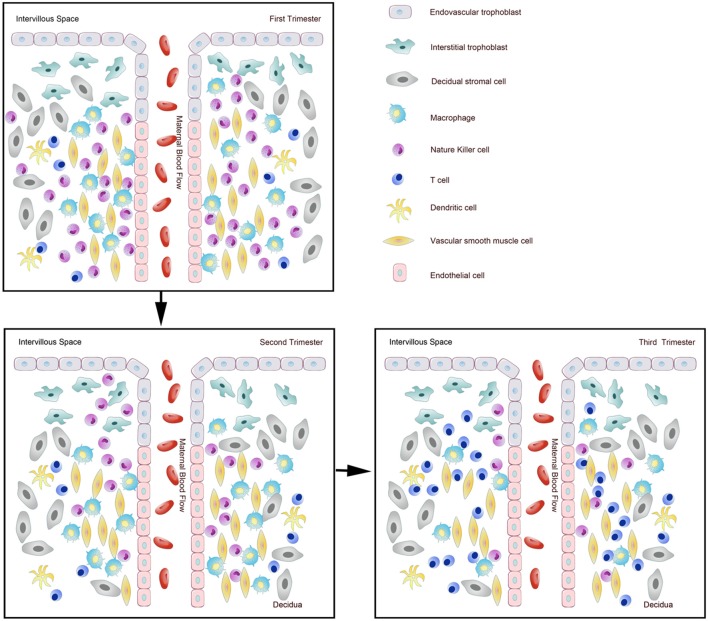
Dynamic changes of immune cells during normal pregnancy at the maternal-fetal interface. Pattern diagram showed the dynamic changes of immune cells at the maternal-fetal interface.

**Figure 2:**
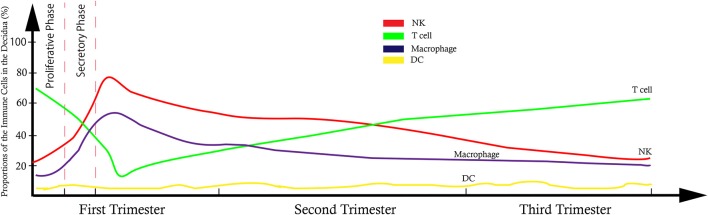
Model of trend line showed the dynamic proportional changes of immune cells at the maternal-fetal interface.

It has been shown that 30–40% of all decidual cells during early pregnancy are leukocytes (6, 8). Various subsets of maternal immune cells constitute the decidual immune system, such as natural killer (NK) cells, macrophages, T cells, B cells, and dendritic cells (DC) (The abbreviated form could be looked up in the Table 1) (6). Fetal trophoblasts, which have direct contact with maternal DSCs and invade the maternal myometrium, are also essential elements in the decidua (4). Previous studies have demonstrated that decidual NK (dNK) cell participate in trophoblast invasion and spiral artery remodeling, while decidual macrophages, as antigen presenting cells, exert phagocytosis, secrete cytokines and modulate the immune balance at the maternal-fetus interface. T cells and DCs have always been considered the critical cells for immune balance regulation. An imbalance between these cells or functional changes may contribute to pathological pregnancy, including preeclampsia (PE), intrauterine growth retardation (IUGR), recurrent spontaneous abortion (RSA), preterm birth, and congenital infection. With the progress of research, placental tissues have been considered a promising source of stem cells for clinical trials (3, 9); thus, it is crucial to understand the physiological state of these immune cell populations in the decidua. However, the factors that influence immune cell composition and activation status in the decidua basalis and parietalis are still poorly characterized. This review covers major types of immune cells in the decidua and presents their proportion changes and functions during different stages of pregnancy (Figures 1, 2), which can help us understand the relationship between the adverse changes and pathological pregnancy.

**Table 1:** Abbreviated form.

Cytotoxic T-lymphocyte antigen 4	CTLA-4
C–C chemokine receptor type 2	CCR2
Decidual natural killer cell	dNK cell
Dendritic cell	DC
Decidual stromal cells	DSCs
DC-specific intercellular adhesion molecule 3-grabbing non-integrin	DC-SIGN
Extracellular matrix	ECM
Extra-villous trophoblasts	EVTs
Fetal growth restriction	FGR
Granulocyte-macrophage-colony-stimulating factor	GM-CSF
Granulocyte-colony-stimulating factor	G-CSF
Intrauterine growth restriction	IUGR
Interferon gamma	IFN-γ
IFN-Inducible protein-10	IP-10
Ig-Like transcripts	ILT
Integrin alpha-X	CD11c
Interleukin	IL
Lipopolysaccharide	LPS
Killer immunoglobulin-like receptors	KIR
Macrophage-colony-stimulating factor	M-CSF
Nitric oxide	NO
Natural killer cell	NK cell
Programmed cell death-1	PD-1
Pre-eclampsia	PE
Periphery blood NK	pbNK
Recurrent spontaneous abortion	RSA
Retinoic acid receptor-related orphan receptor-γt	RORγt
Tumor necrosis factor	TNF
Transforming growth factor	TGF
T-cell immunoglobulin mucin-3	Tim-3
Toll-like receptor	TLR

*This table identifies the full name of the abbreviation in the article*.

## Nature Killer Cells

### NK Cells in Pregnancy

In humans, the typical phenotype of NK cells is CD3^−^CD56^+^, which can be classified into different phenotypes in the peripheral blood. Approximately 90% of peripheral blood NK (pbNK) cells have a CD56^dim^CD16^+^ surface phenotype that represents cytotoxic NK cells, while the remaining 10% possess a CD56^bright^CD16^−^ phenotype, representing cytokine-producing NK cells. Cytokine-producing NK cells are abundant in granules, which contains granzymes/granulysin/perforin, show weak cytotoxicity, and produce a variety of cytokines (4, 6, 10).

In the human endometrium and decidua, prior studies have noted that the prominent NK cells are CD56^bright^CD16^−^. In the last three decades, there has been no definite conclusion on the origin of dNK cells. Several references identified dNK cells as a heterogeneous population. Three different theories have been proposed about the origin of dNK cells. Carlino et al. showed that recruitment of pbNK cells to the uterus is attributed to the accumulation of dNK cells (11). They illustrated that dNK cells and pbNK cells have similar chemokine receptor patterns (very low levels of CCR1 and CCR5) when they contact decidual stromal cells (11). The second, shown by Manaster et al. claims that some progenitor dNK cells might mature from endometrial NK cells in response to pregnancy-associated factors, such as IL-15 or progesterone (6, 12). The third theory suggests that dNK cells may directly differentiate from hematopoietic precursors in the decidua in response to specific stromal factors of the decidual microenvironment (13). The origin of dNK cells may be convoluted, and these three mechanisms may also act concurrently, which indicates that further investigations are necessitated on this subject.

The proportion of NK cells populating the endometrium of non-pregnant women varies with the menstrual cycle. After labeling NK cells with CD56 and CD16, 17% of menstrual blood CD45^+^ lymphocytes were found to be NK cells by flow cytometry (14). King et al. reported the number of endometrial NK cell is low during the follicular phase and early secretory phase, while gradually increasing after ovulation, ultimately, these cells reach the maximum in the late luteal phase (15). Other have found a periovulatory increase (16). The percentage of endometrial NK cells in the stromal compartment rose up to 7.35% in the late secretory phase (17). Reports are conflicting about the number and activity of these cells. Manaster et al. indicated that ~30% of endometrial lymphocytes are NK cells (gated CD56^+^CD3^−^), and the percentage of NK cells remained constant during the menstrual cycle. They showed an increase in NK cell numbers during the secretory phase by immunohistochemistry analysis (12). More studies suggest that the number of NK cells gradually increases with the menstrual cycle. This increase may indicate that the lymphocytes in endometrial increased along with the menstrual cycle. Many sample factors, including the presence of edema, depth from the surface epithelium and site in the uterus can affect endometrial NK numbers (18), which eventually cause the discrepancy of these results.

dNK cells reach the maximum in frequency during first pregnancy, which accounts for more than 70% of decidual leukocytes and then progressively decreases in number and become less granular through gestation, resulting in small amounts at term (19–21). Williams et al. found that CD56^+^ cell numbers in the decidua basalis did not change significantly between the first (45.2 ± 2.8%) and second (48.7 ± 4.0%) trimesters, as well as in the decidua parietalis, while a significant reduction was observed in the third trimester (29.0 ± 3.3%) (22). This result was confirmed in the study of Bulmer et al. who analyzed leukocyte numbers in placental bed biopsies obtained from the termination of normal pregnancy at 8–20 weeks gestation by immunohistochemistry (8). They observed an exciting change that the number of CD56^+^ NK cells which express perforin and granzyme B in the placenta bed biopsies were reduced at 16–20 weeks gestation compared to 8–10 and 12–14 weeks (8). This reduction may reflect the loss of granules. The explanation for this change in the cytoplasm may be a functional alteration of dNK cells as gestation progressing (8).

### The Function of dNK Cells

Previous researchers suggested endometrial NK cells are specialized immature cells since they possess no apparent functional activity and lack the expression of chemokine receptors. They supposed endometrial NK cells shift to dNK cells under the stimulation of IL-15 after conception, a key cytokine in the differentiation of hematopoietic progenitor to NK cells (12).

Most existing studies have focused on the function in dNK cells. Apart from early pregnancy and delivery, specimens from other gestational ages are hard to obtain due to ethical principles. Thus far, the function of dNK cells has not been illustrated clearly, especially their role in middle and late pregnancy.

DNK cells promote embryonic development and are also involved in maintaining the decidual tolerance to embryos. Previous studies have reported that dNK cells participate in trophoblast invasion and spiral artery remodeling during the first trimester. dNK cells regulate the invasion of trophoblasts by producing the chemokines IL-8 and IP-10, which bind to CXCR1 and CXCR3 expressed by invasive EVTs, respectively (23). dNK cells were a major source of VEGF-C, Arg1, Arg2, and TGF-β1 in first trimester and were thought to initiate decidual-associated artery remodeling: readying the vessels for trophoblast infiltration and the final stages of spiral artery remodeling (24). Craven reported that EVTs were absent in the early spiral arteries, which indicated the important role of dNK cells in the reconstruction of spiral arteries (25). A study focusing on dynamic changes in normal murine pregnancy found that dNK cell-derived IFN-γ strongly contributed to initiating uterine vascular modification (26). Apart from the cytokines stated above, dNK cells can produce G-CSF, GM-CSF, M-CSF, TNF-α, which are also involved in successful pregnancy (27, 28). Review by Wallace et al. showed that dNK cells affect EVTs differentiation into an invasive phenotype by secreting factors, such as IL-8 and LIF. Also, dNK promotes invasive EVTs to gain the “endothelial-like” properties (29).

dNK cells are believed to contain limited cytotoxicity to trophoblast cells due to the interaction between specific HLA class I molecules expressed by EVTs and the activating or inhibitory receptors on dNK cells (6). EVTs express the fetal polymorphic classical class Ia MHC-molecule (HLA-C) and non-classical class Ib MHC-molecules (HLA-E, HLA-G) (30–32), but not HLA-A or HLA-B. It is in contrast to most somatic cells (30). dNK cells express a group of receptors, including KIR, ILT, and CD94/NK group 2 (NKG2) A/C/E heterodimers (20, 33, 34). HLA-C antigens can also be classified into two types: HLA-C1 that are less inhibitory to NK cells and HLA-C2 that are more inhibitory (35). KIR receptor genotype can be divided into two forms depending on the presence (A) or absence (B) of activatory KIR receptors (29). Higher risk of pre-eclampsia is associated with the engagement of AA genotype KIR and HLA-C2 (36). The invariant CD94 associates with members of the small NKG2 family to form heterodimers. The CD94/NKG2A receptor binds to the HLA-E molecule to provide an overall inhibitory signal of preventing cell lysis (21, 33, 37). Interestingly, a peptide derived from the leader sequence of HLA-G, which is expressed only by EVT can affect the interaction between HLA-E and its receptor expressed on the dNK cells (21). Inhibitory receptors on dNK cells include LILRB1, KIR2DL4, and CD94/NKG2A (8). NK-activating receptors include NKp46, NKp44, NKp30, NKG2D, and CD94/NKG2C (38). Cytotoxicity is downregulated once engagement occurs between these inhibitory receptors and the ligands on trophoblasts (4). Approximately 40% of dNK cells express the inhibitory receptor LILRB1, which has a high affinity for the dimeric form of HLA-G (4). Besides, some activating receptors of the KIR family are not cytotoxic to EVTs (39). Using single-cell sequencing to analyzing the cell composition of the decidua in the first trimester, Vento-Tormo et al. found the dNK cells could be divided into three subpopulations: dNK1, dNK2, and dNK3. dNK1 cells highly express KIR inhibitory receptors and LILRB1 which suggesting dNK1 cells particularly interact with EVTs (34). Other studies have demonstrated that dNK cell cytotoxicity is inhibited by macrophages through a TGFβ1-dependent mechanism, as purified and exogenously stimulated dNK cells are capable of killing cellular targets (10).

Recently, Fu et al. demonstrated that CD49a^+^Eomes^+^ dNK cells could directly promote fetal growth by producing growth-promoting factors for embryo development before the establishment of the placenta both in human and mice. This subset of dNK cell specifically produced pleiotrophin, osteoglycin, and osteopontin. Deficient in these growth factors ultimately leads to growth restriction caused by abnormal bone development in offspring. Therefore, this study reveals other new functional roles of dNK cells during embryonic development in early pregnancy (40).

Few studies have distinguished NK cell function between 8 and 12 weeks of gestation since notably altered levels of oxygen occur with this period. With the progressing of pregnancy, dNK cells lost granules in the cytoplasm, which indicates that a functional shift is needed at late gestation for labor and parturition (22). Sindram-Trujillo et al. found no difference of CD56^bright^CD16^−^ NK cells and a significant increase of CD56^dim^CD16^+^ NK cells in decidua when compared spontaneous vaginal delivery with elective cesarean section in uncomplicated human term pregnancy (41). This phenomenon indicates a dynamic phenotypical change of NK cells is necessary for spontaneous vaginal delivery.

CD56^bright^CD16^−^ NK cells are essential for the establishment of pregnancy. According to previous studies, it is vital to maintain proper NK cell activation status during each healthy pregnancy. Alterations in decidual NK cell numbers and activation status can cause complications during pregnancy, such as immunologic infertility, recurrent spontaneous abortion, and preeclampsia, but the mechanism is not precise. The relationship between NK cell abnormalities and complications during pregnancy will be described later.

## Macrophages

### Macrophages in Pregnancy

Macrophages are myeloid immune cells and compose a portion of the innate immune system that strategically resides in tissues where they detect, ingest dead cells and debris, antigen process and present and who are phenotypically and functionally heterogeneous (6, 42, 43). Monocytes are generally believed to be the sole precursors of tissue macrophages (44). Circulating short-lived monocytes make up ~5–10% of peripheral blood leukocytes and migrate to various tissues to become macrophages after residing in the peripheral blood for 1–2 days (45). Macrophages are widely distributed and positioned in human tissues.

Either CD14 or CD68 is generally used as a marker to identify uterine macrophages (8). Based on phenotypical and functional characteristics, macrophages used to be categorized into two significant subpopulations: M1 and M2. Induced by IFN-γ and LPS, M1 macrophages are a classically activated/inflammatory type that participates in antigen presentation, pro-inflammatory cytokine and nitric oxide production, and reactive oxygen species. M2 macrophages, induced by interleukin-4 (IL-4), are an alternatively activated/regenerative type that is responsible for immune tolerance and tissue remodeling (46–48). Before blastocyst peri-implantation, decidual macrophages skew toward M1 polarization, while they skew toward a mixed M1/M2 profile after implantation when trophoblast cells begin to invade the uterine myometrium. It is not until the end of placental development that decidual macrophages transform into a predominantly M2 phenotype that protects the fetus and placenta until parturition (45).

Previous studies have demonstrated that the number of macrophages fluctuates during the menstrual cycle. It seems likely that the recruitment of monocytes to the uterus is driven by estrogen and progesterone. After fertilization, monocytes are attracted to the endometrium immediately in response to pro-inflammatory, metabolic, and immune stimuli. Macrophages are the second most abundant endometrial leukocytes population (49). Macrophages are present in the endometrium/decidua and myometrium. Macrophage numbers increase from 1–2% in the proliferative phase to 3–5% in the secretory phase and 6–15% during the menstrual phase (50). However, Biswas et al. observed no significant changes in macrophage numbers during the menstrual cycle (9), as well as in the research of Repnik et al. (51). In the first trimester, macrophages make up ~20–30% of decidual leukocytes at the maternal-fetal interface (52). The percentage of decidual macrophages among leukocytes did not significantly change between early and middle pregnancy, but significantly decrease in the third trimester (22). Research of Repnik et al. showed similar trends when used CD14^+^ as the marker for monocytes in the decidua (51). The difference in the study of Bartmann et al. is CD14^+^ monocytes increase abruptly at the end of early pregnancy and remain stable until term (53).

### The Function of Decidual Macrophages

Endometrial macrophages play an important role in tissue degradation during menstruation, clearing endometrial debris and repairing and reconstructing tissue structure (9, 50). As antigen presenting cells, macrophages can capture antigen from the semi-allogeneic fetus; secrete various cytokines, chemokines, angiogenic growth factors and proteases; and phagocytose, which plays a vital role in homeostasis, host defense, immune responses, tissue development, and repair (44, 45, 49). Utilizing a combination of CCR2 and CD11c, one study recently identified three distinct subsets of decidual macrophages [CCR2^−^CD11c^LO^ (CD11c^low^, ~80%), CCR2^−^CD11c^HI^ (CD11c^high^, ~5%), and CCR2^+^CD11c^HI^ (CD11c^high^, 10–15%)] in early pregnancy by flow cytometry analysis (48). Different distributions were detected among the three subsets, of which CCR2^−^CD11c^HI^ and CCR2^+^CD11c^HI^ macrophages exhibited a polar distribution at the maternal-fetal interface, while CCR2^−^CD11c^LO^ macrophages distribute in the decidua (48). The researchers also found that CCR2^+^CD11c^HI^ macrophages showed pro-inflammatory characteristics, while the CCR2^−^CD11c^HI^ population is suggested to be anti-oxidative and anti-inflammatory (48). Macrophages, gathering in the uterus during the late luteal phase, as well as increased macrophage products, such as MIP-1β, MIF, and CSF-1, are presumed to exert specific roles in the regulation of fertility (50). During the window of implantation, macrophages establish a proinflammatory microenvironment for embryo implantation (50). Previous studies have indicated macrophages are in the vicinity of spiral arteries (49), which lead to disruption and disorganization of vascular smooth muscle cells and endothelial cells even before EVTs are present (54). MMP-7 and -9, which are proteolytic enzymes expressed by macrophages, participate in the disruption and loosening of cohesion between vascular smooth muscle cells layers, representing a loss of integrity of the vascular ECM (54). In these ways, macrophages prepare spiral arteries for further reconstruction by trophoblast cells.

Due to significant secretory production of macrophage in late gestation, which includes IL-1β, IL-6, TNF-α, MMP, and NO (55–58), macrophages are considered as the primary innate immune cells that contribute to the processes of term and preterm labor (58). It has been demonstrated that NO can inhibit the myometrium contraction (59). In mice, the number of macrophages in the uterus at 4 days prior to birth (mid/late gestation) were significantly higher than in non-pregnant controls and decreased to the level of non-pregnant 1 day before delivery (58, 60). NO production increased in the uterus of rats during pregnancy but reduced during the term, which correlates with the change of macrophage (61, 62). These results suggest that the onset of labor due to having a close relationship with a decrease in macrophages and the resultant reduction in NO.

CD14^+^ decidual macrophages are considered to play a prominent role in regulating adaptive T cell responses and innate NK cell responses during the first trimester (63). Decidual macrophages are in close proximity to dNK, which implicating macrophage as potential mediators (7). A functional study demonstrated that interactions between B7-H1 expressed on decidual macrophages and its ligand PD-1 expressed on decidual T cells suppress T cells' IFN-γ production in early pregnancy. This engagement suggested that macrophage participates in maintaining immune balance (64).

Embryo implantation and trophoblast invasion are characterized by a progressive, continuous induction of apoptosis in the maternal tissue surrounding the fetus (65). Histological analysis of normal placental beds indicated that a large number of macrophages localized to the vicinity of apoptotic cells (65). Decidual macrophages can induce apoptosis of damaged cells and remove apoptotic cells prior to the release of their intracellular components, which are antigenically foreign to the maternal immune system and may initiate lethal immunological responses for the fetus (65). Decidual macrophages show a powerful capacity to prevent inflammatory reactions or release of pro-inflammatory cytokines from adjacent cells, which become crucial for the immune balance. Aberrant macrophage numbers and activation may play a role in pregnancy complications, such as preeclampsia, intrauterine growth restriction (IUGR), or preterm birth, which will be discussed later.

## T Cells

### T Lymphocytes in Pregnancy

Many studies have investigated the function of αβ T cells (henceforth referred to simply as T cells) at the maternal-fetus interface over the past three decades. However, the specific features of T cells haven't been fully elucidated, and this is becoming one of the most challenging problems in reproductive immunology. Previous studies have suggested that T cells in the decidua play a vital role in both normal and abnormal pregnancy. T cells can be divided into multiple subpopulations that may possess diverse functions. Some subpopulations of T cells can help EVTs invade the endometrium and promote embryo implantation and placental formation, while others are closely correlated to pregnancy complications, such as PE and RSA. Each T cell antigen is specific because of the rearrangements of the TCRα and TCRβ genes during T cell development in the thymus. Thus, identifying the functions of decidual T cells relies on the comprehension of subset differentiation and antigen specificity (66). T cell subsets can be classified into CD4^+^ T cells, which fall into the categories of Th1, Th2, Th17, and regulatory CD4^+^ T (Treg) cells, and cytotoxic T lymphocytes (CTLs), which are effector CD8^+^ T cells (66).

### The Composition of the Decidual T Cell

Studies have demonstrated that T cells constitute 45–60% of the total endometrial leukocytes in the early proliferative phase, while they decrease in percentage in the secretory phase (44). CD3^+^ T lymphocytes make up ~10–20% of the endometrial stromal leukocyte population in the first trimester. Approximately 30–45% are CD4^+^ T cells, and 45–75% are CD8^+^ T cells (8, 66). Based on the chemokine expression profiles, Th2 and Th17 cells account for 5 and 2% of CD4^+^ T cells in the early pregnant decidua, respectively. Approximately 5–30% of CD4^+^ T cells are Th1 (CCR4-CXCR3^+^CCR6^−^) cell. CD25^hi^ FOXP3^+^ Treg cells comprise ~5% of CD4^+^ T cells (66). In the menstrual cycle and different stages of pregnancy, the proportion and percentage of T lymphocytes in leukocytes varies greatly, but the number of T cell populations does not significantly change (8). In the investigation of Williams et al., CD3^+^CD8^+^ T cells remain constant in number throughout gestation since no significant differences were detected in the number of CD3^+^ and CD8^+^ T cells between the first, second, and third trimesters in the decidua basalis (22). Gomez-Lopez et al. observed a significant increase in the proportion of CD4^+^ T cells at term among leukocytes in the decidua when comparing spontaneous labor to those that had not experienced labor (67). T cells become the primary immune cells of the decidua in the third trimester, mainly due to the decrease of dNK cells (22).

### The Function of Decidual T Cells

The classic model of immune regulation during human pregnancy is that the maternal immune response changes from the inflammatory Th1 cytokine pattern to the Th2 pattern. However, researchers have found that this simple binary classification cannot explain the complex immunological interactions at the maternal-fetus interface (26). In the first trimester, Trophoblast cells produce CKCL16, which interacts with CXCR6 expressed on T cells to attract peripheral T lymphocytes, γδ T cells to the decidua, leading to the formation of a specific maternal-fetus microenvironment interface (68). As the proportions aforementioned, it is thought that decidual T cells become crucial in regulating the placental microenvironment and recognition of fetal antigens rather than invading trophoblastic cells and spiral artery remodeling; the functions of T cells are complicated and remain a controversial issue.

#### Cytotoxic T Lymphocytes

Decidual cytotoxic T Lymphocytes, which are CD8^+^ Effector T cells, have the potential to recognize fetal antigen and lyse trophoblast cells directly via HLA-C on EVTs and indirectly via maternal APCs (69). CD8^+^ T cells express higher Tim-3 and PD-1 in the human decidua than in peripheral blood. Decidual Tim-3^+^PD-1^+^CD8^+^ T cells recognize PD-L1 expressed on EVTs, giving an inhibitory signal, resulting in trophoblast antigen-specific tolerance. Furthermore, EVTs enrich Tim-3^+^PD-1^+^CD8^+^ T cells in an HLA-C dependent manner, which means decidual CD8^+^ T cells can tolerate EVTs (70). Researchers also suggested that the effector memory subset (CD8^+^CD45RA2^−^CCR7^−^) who shows a lower expression of perforin and granzyme B (71) was the dominant population of T cells in the decidua basalis (26). Van Egmond et al. found CD8^+^ T cells in decidual tissue at term was virus-specific and significantly higher than that in periphery blood. All these studies indicated that CTLs at the maternal-fetus interface shows the limited cytotoxic activity, which is controlled by the placental tissue.

#### Effector CD4^+^ T Cells

It is already known that Th1, Th2, and Th17 cells are three main effector CD4^+^ T cell subsets (72). Decidual effector CD4^+^ T cells have the potential to recognize fetal antigen and lyse trophoblast cells; however, this functional response is actively suppressed by local T regulatory cells. Th1 cells are mainly involved in the pathogenicity of organ-specific autoimmune diseases in peripheral tissues (73, 74). They express IFN-γ as their signature cytokine as well as TNF-α that is involved in promoting inflammation. It is generally accepted that Th1 cells potentially contribute to pregnancy pathologies and are the main threat to the fetus since Th1 cells are the primary CD4^+^ T cells that drive surgical allograft rejection (45, 66). Stimulation of TLR3 and TLR7 expressed on CD3^+^ T cells induces the Th1-type cytokine production, resulting in abortion in mice (75). The cytokines produced by Th2 cells, such as IL-4, IL-5, IL-6, IL-10, and IL-13, were demonstrated as having pathophysiological importance in allergic inflammation (74). Previous studies demonstrated that Th2 cells could provide an alternative, less embryotoxic differentiation state in comparison to Th1 cells. The cytokines from Th2 cells could repress Th1 cells differentiation and function (66). Raghupathy et al. have verified that women with healthy pregnancy have a higher Th2 bias, while women with a history of RSA tend to Th1 bias since higher levels of Th2 cytokines IL-6, and IL-10 in healthy pregnancy and higher levels of the Th1 cytokine IFN-γ in RSA were detected in the maternal PBMC when cocultured with irradiated autologous placental cells (76). A Th2-skewing model of the T cells response during healthy pregnancy would minimize the generation of Th1 cells.

Previous studies have demonstrated that Th17 cells, which express the transcription factors RORγt, STAT3, IRF4, and secrete members of the pro-inflammatory IL-17 family, have crucial pathogenic roles in human autoimmune diseases and exert host defense against extracellular bacteria and fungi (45, 74). Th17 cells are derived from naïve T-cell precursors by the polarizing cytokines TGF-β and IL-6 (77). Wu et al. observed a markedly elevated percentage of Th17 cells in the first-trimester decidua compared with the endometrium of the secretory phase (77). A matrigel invasion assay indicated that the interaction between IL-17 receptor expressed on the EVTs and IL-17 promoted trophoblast invasion in a dose-dependent manner, which suggests that Th17 cells may affect the trophoblast function (77). As gestation progresses, the percentage of Th17 cells, as well as other inflammatory cytokines, increases during the third trimester, Th17 cells were thought to participate in the initiation of labor (20).

#### Regulatory CD4^+^ T Cells

Regulatory CD4^+^ T (Treg) cells are a unique subset of CD4^+^ T cells that express high levels of CD25 (CD4^dim^CD25^high^) whose differentiation and function are controlled by X chromosome-encoded transcription factor Foxp3. Treg cells are generated in the thymus or extrathymically, and they constitute ~5–15% of the peripheral CD4^+^ T cells in humans (78–80). Treg cells maintain immune homeostasis by suppressing the activity of other immune cell types and downregulating immune responses after the appropriate and efficiently elimination of invading organisms, preventing autoimmunity, and minimizing tissue destruction by the pathogen (81).

Treg cells can be further classified into two main subsets: natural Treg (nTreg) cells and induced Treg cells (iTreg) (82). The former naturally occurs in the thymus from T-cell precursors and constitute a critical arm of mechanisms of peripheral tolerance, particularly to self-antigens, the latter is generated in secondary lymphoid organs from naïve CD4^+^ T cells upon exposure TGF-β (83) and IL-2 (45, 78, 82, 84). Due to lack of sensitive markers to distinguish these cell types, it's hard to define these two types precisely. Helios is an Ikaros transcription factor family member expressed on Treg cells that is first used as a specific marker to identified nTreg cells (85, 86). However, Helios as a marker to distinguish nTregs from iTregs cells is controversial (87).

In human first trimester decidua, about 4% of the CD4^+^ T cells are CD25^hi^ FOXP3^+^ Treg cells (88, 89), within which ~55% at term in turn is putative Helios^+^ nTReg cells, and 45% is putative Helios^−^ iTReg cells (66). CD4^+^CD25^+^FoxP3^+^ Treg cells in human first trimester decidua increase in healthy pregnant decidua compared with peripheral blood (89, 90). The peripheral CD4^+^CD25^+^ T cells increased to the peak from the first to second trimester (91), then decreased in the third trimester until reaching a low level after post-partum (90).

In mice, studies have shown that most of Tregs in early pregnancy are nTreg cells since they express Helios, indicating that these cells originate in the thymus and are essential at the pre-implantation period. nTreg cells decline soon after implantation when these cells are replaced by peripherally-converted Treg cells (79). Other studies observed a decreased number of CD4^+^CD25^+^Foxp3^+^ T cells in RSA decidua (92), as well as the potency for maternal Tregs (93). These evidences indicated Treg cells population act in preventing miscarriage.

The mechanism by which Treg cells mediate immunological tolerance to alloantigens to maintain successful pregnancy remains to be elucidated. Immune checkpoints have been a significant subject of study in recent years since these checkpoints play a vital role in the maintenance of immune balance, especially in the tumor microenvironment. CTLA4 expressed on the Treg cell surface bind to CD80 or CD86 expressed on antigen-presenting cells, especially DCs, which transmits inhibitory signals to T cells (78, 79). Indoleamine 2,3-dioxygenase (IDO) whose metabolite is toxic to T cells, is expressed by decidual stromal cells macrophages DCs (94, 95). Activated Tregs may stimulate DCs to express IDO through the ligation between CTLA4 and CD80 or CD86. Also, it can upregulate IDO expression on decidual stromal cells by the induction of IFN-γ (94–97). These connections between cells can raise the threshold required for T cell activation, thereby preventing the priming of autoreactive T cells (81).

There is an appropriate balance at the maternal-fetal interface between Th1/Th2/Th17 and Treg cells. This balance is built by different components in the uterus/decidua/fetus to compromise with each, including cytokines secreted by immune cells, such as IL-4 and IL-10, the connection between these cells like CTLA4 and the ligand CD80/CD86. Different mechanisms, such as hormonal imbalance, infections, immune, and autoimmune disorders would break the balance will cause complications during pregnancy.

## Dendritic Cells

### Dendritic Cells in Pregnancy

Known as potent antigen-presenting cells, dendritic cells (DCs) have the unique ability to induce both antigen-specific activation and suppression during the immune response. DCs are less abundant in decidua stromal cells but possess the ability to process and present fetal antigens to decidual T cells (98). DCs in the pregnant human decidua were poorly characterized, although they may be important in regulating activated maternal T cells (99, 100). Uterine DCs are of myeloid origin and originate from several potential progenitors, including bone marrow precursors, blood CD34^+^ stem cells, blood monocytes, and possibly tissue macrophages (8).

It is difficult to distinguish myeloid DCs from macrophages. There is neither single specific marker for decidual DCs nor plasticity between these two populations (45, 100). Approximate 1% of the total decidual immune cells in the first trimester are DCs using lineage-negative and HLA-DR^+^ as a combination marker (101). The number of DCs do not appear to change throughout gestation significantly (8). Miyazaki et al. reported that the percentage of myeloid DCs of total leukocytes in the first-trimester decidua was 1.11 ± 0.26%, markedly higher than that in the peripheral blood (102).

DCs are generally classified into two major subtypes: myeloid CD14^−^CD11c^+^ DCs, which reside in the spleen and lymph nodes, are associated with Th1 polarization and regulate pro-inflammatory responses; and plasmacytoid CD123^+^CD11c^−^ DCs (pDCs), which are found in non-lymphoid peripheral tissues and generate Th2 responses (100, 102). Using TLR9 to stimulate pDCs, Moseman et al. demonstrated pDC mediate the generation of CD4^+^CD25^+^ Treg cells. As stated above, Treg cells are hypo-responsive to secondary alloantigen stimulation and strongly inhibit the proliferation of autologous or allogeneic naive CD4^+^ T cells in an Ag-non-specific manner. These suggested pDCs may play vital roles in the maintenance of immunological tolerance. Myeloid DCs in peripheral blood can migrate into different tissues, such as the endometrium, and survive in an immature state. Human decidual DCs can also be classified into two types: a large number of immature DC-SIGN^+^ DCs, which gradually increase with gestational age, and a small number of CD83^+^ DCs (45). During the menstrual cycle, in the endometrium, immature DC-SIGN^+^ cells can transform into mature CD83^+^ DCs by exposure to antigen or inflammatory cytokines, which suggest that DCs are involved in the uterus defense against pathogens (103). Kammerer et al. reported that decidual DC-SIGN^+^ cells *in situ* and *in vitro* exhibit similar functions and phenotypes as immature DCs and these cells could mature in cell culture (104, 105).

### The Function of Decidual Dendritic Cells

The uterine DC population at the time of implantation might be the greatest immunological threat to the fetus since DCs are specific APC. This observation suggests that the processes of decidualization of the endometrium and placental formation are associated with a decrease in CD83^+^ cells and an increase in DC-SIGN^+^ DCs. DC-SIGN^+^ DCs, by ingesting fetal antigens, further mature into CD83^+^ cells and migrate to secondary lymphoid organs to stimulate T cell populations. DC-SIGN^+^ DCs are thought to be plastic in their ability to promote T helper cell responses. During decidualization and embryo implantation, DC-SIGN^+^ DCs recruit NK cells into the endometrium by secreting IL-15 and upregulate the expression of CD56 on NK cells (106). Several studies have elucidated the cross-talk between dNK cells and dDCs during a healthy pregnancy. In a study of mice, Blois et al. reported that murine NK and DC interactions promote a tolerogenic microenvironment and influence the proliferation of uterine stromal cells at the maternal-fetus interface by downregulating the activation markers expressed on dNK cells and dDCs. The interaction is subject to modulation by trophoblast cells *in vitro* (107).

Different subpopulations of DCs have been described as helping to tolerate pregnancy. In the absence of stimulation signals or upon exposure to anti-inflammatory conditions, such as IL-10, progesterone, human chorionic gonadotropin, or estradiol, DCs transformed into tolerogenic DCs (108). Tolerant DCs exert an inhibitory phenotype and produce anti-inflammatory cytokines that prevent T cell activation (109). Subtypes of myeloid DCs (myeloid type 1 and myeloid type 2) were found in the decidua of early pregnancy. Due to the lack of Fc receptors, myeloid type 2 cells recognize fetal antigens in the trophoblast and thus exert an immune tolerance effect (110).

### Specific Immune Protective Mechanisms at the Maternal-Fetus Interface

The placenta is not a typical graft, since proteins derived from HLA genes are not expressed co-dominantly on trophoblast cell membranes, unlike somatic cells. The EVTs display a unique pattern of class Ia HLA-C and the non-classical HLA class Ib molecules, HLA-E, HLA-F, and HLA–G (111–113). HLA-G, HLA-C, and HLA-F are expressed by first trimester EVTs, and, as gestation proceeds, their expression weakens and becomes intracellular. HLA-E is expressed by the EVTs only in the first trimester (112).

HLA-G and HLA-E inhibit immune responses by interacting with leukocyte inhibitory receptors (LIR) on macrophages and NK cells and with T cell receptors on CD8^+^ cells. As the consequences of these interactions, the killer functions of NK cells and macrophages were dampened (114, 115). HLA-G may also activate pathways in decidual NK cells, macrophages, and T cells that promote placentation. EVTs instruct APCs to become tolerogenic DC which secreting IL-10 and promoting the induction of a variety of Tregs by expressing and secreting HLA-G, and releasing IL-10 (116). It is well-reviewed by Gregori et al. about HLA-G at the maternal-fetus interface. APCs expressing either a soluble or membrane-bound form of HLA-G repress T cell alloproliferation via Fas/Fas ligand (FasL) pathway.

Other mechanisms include the B7 family, IDO, TNF superfamily etc. Interactions between villous and EVTs expressed PD-1 (B7H1; CD279) and PDL-1 (CD274) expressed by maternal lymphocytes promote Tregs development and function and inhibit Th17 cells (117). These molecules, expressed as membrane and soluble forms, could kill activated immune cells that targeting the trophoblast by apoptotic signals which were transduced by specific receptors on activated leukocytes.

Uterine changes during pregnancy contribute to maternal immune adaptation, including relative proportions of leukocyte subsets, phenotypic and functional changes, induction of immunomodulatory molecules and changes in cytokine profiles across gestation. Different causes alter placental function and mechanisms of immune tolerance and can disrupt the normal trophoblast-maternal immune system crosstalk, resulting in adverse pregnancy outcomes, such as recurrent spontaneous abortion, preterm labor and preeclampsia.

### Immune Cells in Pathological Pregnancy

#### Pre-Eclampsia

PE, a hypertensive disorder of pregnancy, has significant maternal and fetal morbidity and mortality worldwide. It is a multisystemic disorder relating to the imbalance of the local immune microenvironment of the maternal-fetus interface (118). The initiative characteristic of PE is insufficient EVTs infiltration and deficient spiral artery remodeling, leading to a placental ischemic microenvironment with a resultant increase in oxidative stress (119). Preeclampsia is defined by the onset of hypertension and proteinuria after 20 weeks' gestation. Preeclampsia is subdivided into early-onset (start pre-34 weeks) and late-onset preeclampsia (start after 34 weeks). The early-onset preeclampsia has close relation with impaired trophoblast invasion and spiral arteries remodeling as well as IUGR.

Studies investigating decidual macrophage and NK cells number during PE have shown varying results. The previous study observed no differences, increased or decreased in the number of CD56^+^ dNK cells in women with PE (120–123). dNK numbers decreased (120, 121) or increased (123) in the subgroup of IUGR in the presence or absence of PE (124). However, no significant difference was observed in the distribution of NK cell in the decidua in relation to the severity of pre-eclampsia (123). The number of decidual macrophages in PE increased or decreased in comparison with the normal group (8, 123). Decidual macrophage numbers in the subgroup of PE with fetus IUGR are greater than those without IUGR, even though the difference was not significant (123). Compared with normal pregnancy, the number of M1 macrophages increased, and M2 macrophages decrease in PE and RSA patients' decidua (125, 126), which indicates M1 leads to inflammation of the maternal-fetal interface microenvironment. Numbers of macrophages around the spiral arteries increased in the placental bed of pre-eclamptic patients as compared with that of healthy control women (127).

The number of CD3^+^ and CD8^+^ T lymphocytes in decidua was significantly higher in PE group than normal control (128). Abundant evidence suggests that predominance of Th1-type immunity and pro-inflammatory cytokines are critical for the development of this disease (129). Reviewed by Osborne et al. showed lots of studies had found increased Th17 cells or an increase in the Th17/Tregs ratio in pre-eclampsia women compared to normotensive or non-pregnant subjects when measured in peripheral blood, decidual tissue, or umbilical cord blood (130). No distinct changes in DCs were observed in pre-eclamptic, growth-restricted pregnancies, and RSA patient compared to normal controls (131, 132) while DCs in the decidua of patients with HELPP syndrome increased compared to age-matched controls (133).

Previous studies have also shown that the function of decidual immune cells in preeclampsia patients has changed. The mismatch between dNK cells' KIR-AA genotype receptor and the EVTs' HLA-C2 allotype ligand could lead to strong inhibitory signals to dNK cells. This was more frequent in women with complication of pregnancy associated with poor placentation (119). Zhang et al. identified increased populations of dNK, Tregs, and higher levels of TGFβ1 in the decidua of pre-eclampsia compared to normal control. They suggested increased decidual TGFβ1 secreted by the Tregs suppressed the cytotoxic and angiogenic function of dNK cells, leading to defective placental formation associated with the onset of preeclampsia (134). *In vitro* study, researchers showed that activated macrophages around uterine vessels could inhibit trophoblast invasion and spiral artery remodeling. This effect may indicate the macrophage's importance in the etiology of preeclampsia (135). DC did not directly inhibit trophoblast invasion (136). Huang et al. demonstrated the inflammatory microenvironment of pre-eclampsia could promote dendritic cell to infiltrate to the maternal-fetus interface. Considering the versatility in mediating immunity and tolerance of DC, this study suggests that DC may be involved in the pathogenesis of pre-eclampsia or the prevention in subsequent pregnancies (136). Other researchers have found the maternal blood flow resistance increases in PE and RSA patients. In women with high uterine arterial resistance index, dNK cells were less able to promote trophoblast invasion *in vitro* studies than normal control (137).

Interruption of the normal net connection between dNK cells, trophoblast cells, and other decidual cells cause insufficient invasion of trophoblast cells, resulting in inadequate remodeling of uterine spiral arterioles, which is one of the primary pathogenesis of preeclampsia. Results from previous studies are mixed, which most did not distinguish early/later onset or mild/severe or term/preterm PE. Also, due to the different location of the sample and the method of experimentation, the results of specific changes in dNK cells in PE are inconsistent. Most of the PE occurs in the third trimester, but dNK cells play a role in remodeling blood vessels in early pregnancy. It is impossible to harvest the sample of the first-trimester decidua of preeclampsia. In addition to the uterine arterial resistance index, a good indicator for early prediction of preeclampsia is still lacking. The early pathogenesis of preeclampsia still needs to be further investigated.

#### Recurrent Spontaneous Abortion

Recurrent spontaneous abortion is one of the most frustrating and challenging areas in reproductive medicine. Current evidence-based diagnostic and treatment strategies are few due to the etiology is often unknown. Both autoimmune and alloimmune mechanisms have been proposed that been implicated in the onset of RSA. Of particular importance is antiphospholipid syndrome. APS is the only immune condition in which pregnancy loss is a diagnose criteria for the disease.

Based on the current evidence, whether the number or percentage of NK cell increases in RSA has not been clarified. King et al. reported that CD56^+^ dNK cells increased in the decidua of women with RSA (138). In contrast, dNK cells, expressed as percentages, showed no difference in women with RSA compared with controls (139). Analysis of the leukocytes isolated from non-pregnant women luteal endometrium or women who suffered the URSA did show the phenotype of NK shifting from CD56^bright^CD16^−^ to CD56^dim^CD16^+^ (140). This shifting may represent a transformation in the function of dNK cells from low cytotoxicity to high cytotoxicity. And this change may participate in the onset of RSA. Over-remodeling of the decidual blood vessels during early pregnancy may lead to local oxidative stress, which may also be potential pathogenesis of RSA. Since maturation and differentiation of blood vessels are also crucial for the resistance of blood flow (141).

Guenther et al. found that CD68^+^ macrophages and apoptosis increased significantly in the RSA group than normal control (142, 143). They also found that the percentage of FasL^+^/CD68^+^ cells was markedly higher in the RSA than normal pregnancy group, since FasL has already been reported to be involved in RSA pathophysiology (125, 143). Spontaneous abortion and unexplained RSA decidua are abundant in M1 macrophages, while M2 macrophages in the luteal phase and normal pregnancy decidua are significantly higher (126). Immunosuppressive cytokines (IL10) decreased, and costimulatory molecules (CD80 and CD86) increased in the decidual of URSA patients compared with normal control (144). Co-culture of macrophages with Treg cells results in increased IL10 production, which indicated the regulatory capacity of Treg cells in the URSA (144). The activity and number of macrophages in decidual from URSA polarize toward the M1 phenotype. The inflammatory microenvironment leads to a marked increase in apoptosis, as well as cell debris. Macrophages whose function is impaired can't phagocytose these cell debris. It may eventually lead to inflammatory immunity, endangering embryo safety, and causing miscarriage.

The balance between Th17 and Treg cells and the interaction with many other types of cells form the complex and dynamic networks to maintain homeostasis. Due to the foreign fetal antigens challenge the maternal immune system, Treg cells overwhelm Th17 cells in pregnancy to ensure the balance. Previous studies have found the elevated proportions of Th17 cells and the cytokines they produce, including IL-17 and IL-23, and decreased proportions of Treg cells in human decidua during early pregnancy when comparing RSA with normal pregnancy (93, 130). The clinical applying of intravenous immunoglobulin to address a Th17/Treg imbalance in RSA patients improved pregnancy outcomes (145), even the evidence was inconclusive (146). These all suggest that Th17 and Th1 cells response dominate Treg cells in these pregnant complications (147, 148). Review by Rahimzadeh et al. indicated there were some discrepancies between the results reported by different authors. However, the results were more consistent if similar markers were used to differentiate Tregs originating from the same biopsies (129). Taken together, aberrant Treg cells performance is involved in the pathophysiology of RSA since Treg cells' protective groundwork to maintain immune tolerance.

Research on DCs in RSA patient is few. Immature DCs are mildly upregulated or downregulated upon maturation due to different markers being used (131). Higher CD83^+^ mature DCs and lower CD1a^+^ immature DCs in the decidual stroma was observed from RSA than normal pregnancy (149). Tirado-Gonzalez et al. also observed a marked reduction of DC-SIGN^+^ cells in RSA compared to normal decidua (150). DCs are the most potent antigen presenting cells. The maturation of decidual DCs may participate in the pathogenesis of RSA.

Other pregnancy morbidities including preterm birth (58), chorioamnionitis, cholestasis of pregnancy, and gestational diabetes, were reported that are known to have an inflammatory component and have also been associated with the increased portion of Th17 cells in pregnancy, which have been reviewed by Osborne et al. (130).

## Concluding Remarks

The maternal-fetus interface fills with a variety of components, including decidua/placenta/chorion-amnion. The decidua consists of immune cells, decidual stromal cells, and trophoblast cells which interact with each other to exert delicate functions to maintain a successful pregnancy (Table 2). Decidual NK cells are the predominant immune cells in the human decidua in the first trimester, whereas macrophages are second abundant. In contrast, T cells are less abundant, and DCs are relatively scarce. Complicated interactions between each type of cells to balance maternal-fetal crosstalk have pivotal roles to ensure the survival of a semi-allogeneic fetus. As pregnancy progress, these immune cells fluctuate dynamically to content the gestation requests. Crosstalk in maternal-fetus interface would be a break if dysregulation of these immune cells, eventually jeopardizing the immune imbalance, including insufficient trophoblast invasion, defective decidual vascular remodeling, inadequate maternal tolerance, and an impaired defensive response, which may subsequently cause spontaneous abortion or pregnancy complications. Much signs of progress have been made during the past three decades, while many mysteries haven't yet to be investigated.

**Table 2:** Comparison of decidual leukocyte populations between decidua in the first, second, and third trimesters of pregnancy.

**Cell type**	**Marker**	**Stage of pregnancy**			**Biopsy**	**Method**	**References**
		**First trimester (%)**	**Second trimester (%)**	**Third trimester (%)**			
NK	CD56	84.5 (77.8–88.5)	50.4 (37.9–58.1)	Remain stable	Decidua	FACS	Bartmann et al. (53)
	CD56	45.2 ± 2.8	48.7 ± 4.0	29.0 ± 3.3	Decidua basalis	Immunohistochemical	Williams et al. (120)
Macrophage	CD14s	34.4 ± 2.8	33.3 ± 1.9	17.3 ± 1.2	Decidua Basalis	Immunohistochemical	Williams et al. (120)
	CD14	9.1 (5.0–14.0)	18.2 (12.9–24.2)	Remain stable	Decidua	FACS	Bartmann et al. (53)
Th17 CD4^+^ T cell	IL-17			0.31 ± 0.06	Periphery Blood	Flow cytometry	Santner-Nanan et al. (151)
Treg	CD4^+^CD25^high^			3.12 ± 0.26	Peripheral blood	Flow cytometry	Santner-Nanan et al. (151)
	CD4^+^CD127^low^CD25^+^			6.98 ± 0.42	Peripheral blood	Flow cytometry	Santner-Nanan et al. (151)
	CD4^+^Foxp3^+^			6.26 ± 0.32	Peripheral blood	Flow cytometry	Santner-Nanan et al. (151)
αβ T cells		8.2 (5.4–11.0)	/	26.7 (20.7–39.7)	Decidua	FACS	Bartmann et al. (53)
CD8^+^T cells		17.3 (12.4–23.9)	17.3 (12.4–23.9)	24.6 (20.5–32.6)	Decidua	FACS	Bartmann et al. (53)
CD8^+^T cells		31.7 ± 3.1	27.0 ± 4.6	34.8 ± 5.5	Decidua basalis	Immunohistochemical	Williams et al. (120)
CD3^+^T cells		34.9 ± 3.7	34.6 ± 4.9	40.3 ± 6.8	Decidua basalis	Immunohistochemical	Williams et al. (120)

*This table describes the proportions of major leukocytes in the decidua during different stages of pregnancy. NK cells are the most abundant in the first and second trimester, where they participate in trophoblast invasion and spiral arterial remodeling, and they decrease as pregnancy progresses. Macrophages are the second major cells in the maternal-fetus interface in the first trimester, and they are stable throughout gestation. T cells have different subtypes that can play totally different functions in a successful pregnancy. The table describes the subsets of T cells and the dynamic changes in their proportions during pregnancy. Studies on the dynamic changes of DC are rare, and we do not provide information on the dynamic changes of DC*.

Different researchers used different methods (flow cytometry studies or immunohistochemical etc.) as well as different antibodies and different biopsies, which may explain inconsistent results. The cellular composition of the maternal-fetal interface was previously described by flow cytometry and immunohistochemistry. As technology advances, emerging technologies can analyze the microenvironment of the maternal-fetal interface from a deeper level. With the advancement of medicine, more and more couples with impaired fertility hope to harbor their offspring. Therefore, understanding the normal physiology of pregnancy will help to reveal the pathogenesis of pregnancy complications. Thus, the treatment of the disease is more meaningful.

## Author Contributions

All authors listed have made a substantial, direct and intellectual contribution to the work, and approved it for publication. FY wrote this review. FY, QZ, and LJ modified this review.

### Conflict of Interest

The authors declare that the research was conducted in the absence of any commercial or financial relationships that could be construed as a potential conflict of interest.

## References

[1] Hsu P Nanan RK. Innate and adaptive immune interactions at the fetal-maternal interface in healthy human pregnancy and pre-eclampsia. Front Immunol. (2014) 5:125. 10.3389/fimmu.2014.00125 24734032 PMC3975095

[2] Glover LE Crosby D Thiruchelvam U Harmon C Chorcora CN Wingfield MB. Uterine natural killer cell progenitor populations predict successful implantation in women with endometriosis-associated infertility. Am J Reprod Immunol. (2018) 79:e12817. 10.1111/aji.12817 29380456

[3] Solders M Gorchs L Gidlof S Tiblad E Lundell AC Kaipe H. Maternal adaptive immune cells in decidua parietalis display a more activated and coinhibitory phenotype compared to decidua basalis. Stem Cells Int. (2017) 2017:8010961. 10.1155/2017/8010961 29317870 PMC5727765

[4] Le Bouteiller P Bensussan A. Up-and-down immunity of pregnancy in humans. F1000Res. (2017) 6:1216. 10.12688/f1000research.11690.1 28781765 PMC5531158

[5] Schumacher A Sharkey DJ Robertson SA Zenclussen AC Immune cells at the fetomaternal interface: how the microenvironment modulates immune cells to foster fetal development. J Immunol. (2018) 2012:325–34. 10.4049/jimmunol.1800058 29987001

[6] Faas MM de Vos P. Uterine NK cells and macrophages in pregnancy. Placenta. (2017) 56:44–52. 10.1016/j.placenta.2017.03.001 28284455

[7] Hsu P Santner-Nanan B Dahlstrom JE Fadia M Chandra A Peek M Altered decidual DC-SIGN+ antigen-presenting cells and impaired regulatory T-cell induction in preeclampsia. Am J Pathol. (2012) 1816:2149–60. 10.1016/j.ajpath.2012.08.032 23063509

[8] Bulmer JN Williams PJ Lash GE Immune cells in the placental bed. Int J Dev Biol. (2010) 542–3:281–94. 10.1387/ijdb.082763jb 19876837

[9] Biswas Shivhare S Bulmer JN Innes BA Hapangama DK Lash GE. Menstrual cycle distribution of uterine natural killer cells is altered in heavy menstrual bleeding. J Reprod Immunol. (2015) 112:88–94. 10.1016/j.jri.2015.09.001 26398782

[10] Co EC Gormley M Kapidzic M Rosen DB Scott MA Stolp HA Maternal decidual macrophages inhibit NK cell killing of invasive cytotrophoblasts during human pregnancy. Biol Reprod. (2013) 886:155 10.1095/biolreprod.112.099465 PMC4070869 23553431

[11] Carlino C Stabile H Morrone S Bulla R Soriani A Agostinis C Recruitment of circulating NK cells through decidual tissues: a possible mechanism controlling NK cell accumulation in the uterus during early pregnancy. Blood. (2008) 1116:3108–15. 10.1182/blood-2007-08-105965 18187664

[12] Manaster I Mizrahi S Goldman-Wohl D Sela HY Stern-Ginossar N Lankry D Endometrial NK cells are special immature cells that await pregnancy. J Immunol. (2008) 1813:1869–76. 10.4049/jimmunol.181.3.1869 18641324

[13] Vacca P Vitale C Montaldo E Conte R Cantoni C Fulcheri E CD34+ hematopoietic precursors are present in human decidua and differentiate into natural killer cells upon interaction with stromal cells. Proc Natl Acad Sci USA. (2011) 1086:2402–7. 10.1073/pnas.1016257108 PMC3038730 21248224

[14] Ivarsson MA Stiglund N Marquardt N Westgren M Gidlof S Bjorkstrom NK Composition and dynamics of the uterine NK cell KIR repertoire in menstrual blood. Mucosal Immunol. (2017) 102:322–31. 10.1038/mi.2016.50 27271316

[15] King A Wellings V Gardner L Loke YW Immunocytochemical characterization of the unusual large granular lymphocytes in human endometrium throughout the menstrual cycle. Human Immunol. (1989) 243:195–205. 10.1016/0198-8859(89)90060-8 2925453

[16] Lee S Kim J Jang B Hur S Jung U Kil K Fluctuation of peripheral blood T, B, and NK cells during a menstrual cycle of normal healthy women. J Immunol. (2010) 1851:756–62. 10.4049/jimmunol.0904192 20530263

[17] Drury JA Parkin KL Coyne L Giuliani E Fazleabas AT Hapangama DK The dynamic changes in the number of uterine natural killer cells are specific to the eutopic but not to the ectopic endometrium in women and in a baboon model of endometriosis. Reprod Biol Endocrinol. (2018) 161:67 10.1186/s12958-018-0385-3 PMC6052567 30021652

[18] Moffett A Shreeve N First do no harm: uterine natural killer (NK) cells in assisted reproduction. Human Reprod. (2015) 307:1519–25. 10.1093/humrep/dev098 PMC4472320 25954039

[19] Gaynor LM Colucci F. Uterine natural killer cells: functional distinctions and influence on pregnancy in humans and mice. Front Immunol. (2017) 8:467. 10.3389/fimmu.2017.00467 28484462 PMC5402472

[20] Taylor EB Sasser JM Natural killer cells and T lymphocytes in pregnancy and pre-eclampsia. Clin Sci. (2017) 13124:2911–7. 10.1042/CS20171070 29222389

[21] Moffett-King A Natural killer cells and pregnancy. Nat Rev Immunol. (2002) 29:656–63. 10.1038/nri886 12209134

[22] Williams PJ Searle RF Robson SC Innes BA Bulmer JN Decidual leucocyte populations in early to late gestation normal human pregnancy. J Reprod Immunol. (2009) 821:24–31. 10.1016/j.jri.2009.08.001 19732959

[23] Hanna J Goldman-Wohl D Hamani Y Avraham I Greenfield C Natanson-Yaron S. Decidual NK cells regulate key developmental processes at the human fetal-maternal interface. Nat Med. (2006) 12:1065. 10.1038/nm1452 16892062

[24] Lash GE Schiessl B Kirkley M Innes BA Cooper A Searle RF Expression of angiogenic growth factors by uterine natural killer cells during early pregnancy. J Leukoc Biol. (2006) 803:572–80. 10.1189/jlb.0406250 16816146

[25] Craven CM Morgan T Ward K Decidual spiral artery remodelling begins before cellular interaction with cytotrophoblasts. Placenta. (1998) 194:241–52. 10.1016/S0143-4004(98)90055-8 9639319

[26] Ashkar AA Di Santo JP Croy BA Interferon gamma contributes to initiation of uterine vascular modification, decidual integrity, and uterine natural killer cell maturation during normal murine pregnancy. J Exp Med. (2000) 1922:259–70. 10.1084/jem.192.2.259 PMC2193246 10899912

[27] Saito S Nishikawa K Morii T Enomoto M Narita N Motoyoshi K Cytokine production by CD16-CD56bright natural killer cells in the human early pregnancy decidua. Int Immunol. (1993) 55:559–63. 10.1093/intimm/5.5.559 7686393

[28] Robertson SA GM-CSF regulation of embryo development and pregnancy. Cytokine Growth Factor Rev. (2007) 183–4:287–98. 10.1016/j.cytogfr.2007.04.008 17512774

[29] Wallace AE Fraser R Cartwright JE Extravillous trophoblast and decidual natural killer cells: a remodelling partnership. Hum Reprod Update. (2012) 184:458–71. 10.1093/humupd/dms015 PMC3373213 22523109

[30] Apps R Murphy SP Fernando R Gardner L Ahad T Moffett A Human leucocyte antigen (HLA) expression of primary trophoblast cells and placental cell lines, determined using single antigen beads to characterize allotype specificities of anti-HLA antibodies. Immunology. (2009) 1271:26–39. 10.1111/j.1365-2567.2008.03019.x PMC2678179 19368562

[31] Sharkey AM Xiong S Kennedy PR Gardner L Farrell LE Chazara O Tissue-specific education of decidual NK cells. J Immunol. (2015) 1957:3026–32. 10.4049/jimmunol.1501229 PMC4574523 26320253

[32] Parham P Moffett A Variable NK cell receptors and their MHC class I ligands in immunity, reproduction and human evolution. Nat Rev Immunol. (2013) 132:133–44. 10.1038/nri3370 PMC3956658 23334245

[33] King A Hiby SE Gardner L Joseph S Bowen JM Verma S. Recognition of trophoblast HLA class I molecules by decidual NK cell receptors—a review. Placenta. (2000) 21:S81–5. 10.1053/plac.1999.0520 10831129

[34] Vento-Tormo R Efremova M Botting RA Turco MY Vento-Tormo M Meyer KB Single-cell reconstruction of the early maternal-fetal interface in humans. Nature. (2018) 5637731:347–53. 10.1038/s41586-018-0698-6 PMC7612850 30429548

[35] Sargent IL Borzychowski AM Redman CW NK cells and pre-eclampsia. J Reprod Immunol. (2007) 761–2:40–4. 10.1016/j.jri.2007.03.009 17482272

[36] Hiby SE Walker JJ O'Shaughnessy K M Redman CW Carrington M Trowsdale J Combinations of maternal KIR and fetal HLA-C genes influence the risk of preeclampsia and reproductive success. J Exp Med. (2004) 2008:957–65. 10.1084/jem.20041214 PMC2211839 15477349

[37] Petrie EJ Clements CS Lin J Sullivan LC Johnson D Huyton T CD94-NKG2A recognition of human leukocyte antigen (HLA)-E bound to an HLA class I leader sequence. J Exp Med. (2008) 2053:725–35. 10.1084/jem.20072525 PMC2275392 18332182

[38] Manaster I Mandelboim O. The unique properties of human NK cells in the uterine mucosa. Placenta. (2008) 29(Suppl. A):S60–6. 10.1016/j.placenta.2007.10.006 18039547

[39] Le Bouteiller P Blaschitz A. The functionality of HLA-G is emerging. Immunol Rev. (1999) 167:233–44. 10.1111/j.1600-065X.1999.tb01396.x 10319265

[40] Fu B Zhou Y Ni X Tong X Xu X Dong Z Natural killer cells promote fetal development through the secretion of growth-promoting factors. Immunity. (2017) 476:1100–13.e6. 10.1016/j.immuni.2017.11.018 29262349

[41] Sindram-Trujillo AP Scherjon SA van Hulst-van Miert PP Kanhai HH Roelen DL Claas FH Comparison of decidual leukocytes following spontaneous vaginal delivery and elective cesarean section in uncomplicated human term pregnancy. J Reprod Immunol. (2004) 621–2:125–37. 10.1016/j.jri.2003.11.007 15288188

[42] Varol C Mildner A Jung S. Macrophages: development and tissue specialization. Ann Rev Immunol. (2015) 33:643–75. 10.1146/annurev-immunol-032414-112220 25861979

[43] Gentek R Molawi K Sieweke MH Tissue macrophage identity and self-renewal. Immunol Rev. (2014) 2621:56–73. 10.1111/imr.12224 25319327

[44] Gomez-Lopez N Guilbert LJ Olson DM Invasion of the leukocytes into the fetal-maternal interface during pregnancy. J Leukoc Biol. (2010) 884:625–33. 10.1189/jlb.1209796 20519637

[45] Liu S Diao L Huang C Li Y Zeng Y Kwak-Kim JYH. The role of decidual immune cells on human pregnancy. J Reprod Immunol. (2017) 124:44–53. 10.1016/j.jri.2017.10.045 29055791

[46] Wheeler KC Jena MK Pradhan BS Nayak N Das S Hsu CD VEGF may contribute to macrophage recruitment and M2 polarization in the decidua. PLoS ONE. (2018) 131:e0191040 10.1371/journal.pone.0191040 PMC5764356 29324807

[47] Zhou D Huang C Lin Z Zhan S Kong L Fang C Macrophage polarization and function with emphasis on the evolving roles of coordinated regulation of cellular signaling pathways. Cell Signal. (2014) 262:192–7. 10.1016/j.cellsig.2013.11.004 24219909

[48] Jiang X Du MR Li M Wang H. Three macrophage subsets are identified in the uterus during early human pregnancy. Cell Mol Immunol. (2018) 15:1027–37. 10.1038/s41423-018-0008-0 29618777 PMC6269440

[49] Ning F Liu H Lash GE The Role of Decidual Macrophages During Normal and Pathological Pregnancy. Am J Reprod Immunol. (2016) 753:298–309. 10.1111/aji.12477 26750089

[50] Thiruchelvam U Dransfield I Saunders PT Critchley HO The importance of the macrophage within the human endometrium. J Leukoc Biol. (2013) 932:217–25. 10.1189/jlb.0712327 23108100

[51] Repnik U Tilburgs T Roelen DL van der Mast BJ Kanhai HH Scherjon S Comparison of macrophage phenotype between decidua basalis and decidua parietalis by flow cytometry. Placenta. (2008) 295:405–12. 10.1016/j.placenta.2008.02.004 18353434

[52] Faas MM De Vos P. Innate immune cells in the placental bed in healthy pregnancy and preeclampsia. Placenta. (2018). 10.1016/j.placenta.2018.04.012 29748088

[53] Bartmann C Segerer SE Rieger L Kapp M Sutterlin M Kammerer U Quantification of the predominant immune cell populations in decidua throughout human pregnancy. Am J Reprod Immunol. (2014) 712:109–19. 10.1111/aji.12185 24330065

[54] Smith SD Dunk CE Aplin JD Harris LK Jones RL Evidence for immune cell involvement in decidual spiral arteriole remodeling in early human pregnancy. Am J Pathol. (2009) 1745:1959–71. 10.2353/ajpath.2009.080995 PMC2671283 19349361

[55] Hibbs JB Jr Taintor RR Vavrin Z Rachlin EM Nitric oxide: a cytotoxic activated macrophage effector molecule. Biochem Biophys Res Commun. (1988) 1571:87–94. 10.1016/S0006-291X(88)80015-9 3196352

[56] Pavlov O Pavlova O Ailamazyan E Selkov S Characterization of cytokine production by human term placenta macrophages *in vitro*. Am J Reprod Immunol. (2008) 606:556–67. 10.1111/j.1600-0897.2008.00657.x 18853988

[57] Huang WC Sala-Newby GB Susana A Johnson JL Newby AC Classical macrophage activation up-regulates several matrix metalloproteinases through mitogen activated protein kinases and nuclear factor-kappaB. PLoS ONE. (2012) 78:e42507 10.1371/journal.pone.0042507 PMC3411745 22880008

[58] Gomez-Lopez N Louis D St. Lehr MA Sanchez-Rodriguez EN Arenas-Hernandez M Immune cells in term and preterm labor. Cell Mol Immunol. (2014) 116:571–81. 10.1038/cmi.2014.46 PMC4220837 24954221

[59] Izumi H Yallampalli C Garfield RE Gestational changes in L-arginine-induced relaxation of pregnant rat and human myometrial smooth muscle. Am J Obstet Gynecol. (1993) 1695:1327–37. 10.1016/0002-9378(93)90301-X 8238202

[60] Mackler AM Iezza G Akin MR McMillan P Yellon SM Macrophage trafficking in the uterus and cervix precedes parturition in the mouse. Biol Reprod. (1999) 614:879–83. 10.1095/biolreprod61.4.879 10491619

[61] Buhimschi I Ali M Jain V Chwalisz K Garfield RE Differential regulation of nitric oxide in the rat uterus and cervix during pregnancy and labour. Hum Reprod. (1996) 118:1755–66. 10.1093/oxfordjournals.humrep.a019481 8921128

[62] Dong YL Gangula PR Yallampalli C Nitric oxide synthase isoforms in the rat uterus: differential regulation during pregnancy and labour. J Reprod Fertil. (1996) 1072:249–54. 10.1530/jrf.0.1070249 8882292

[63] Houser BL Decidual macrophages and their roles at the maternal-fetal interface. Yale J Biol Med. (2012) 851:105–18. PMC3313525 22461749

[64] Sayama S Nagamatsu T Schust DJ Itaoka N Ichikawa M Kawana K Human decidual macrophages suppress IFN-gamma production by T cells through costimulatory B7-H1:PD-1 signaling in early pregnancy. J Reprod Immunol. (2013) 1002:109–17. 10.1016/j.jri.2013.08.001 24045115

[65] Abrahams VM Kim YM Straszewski SL Romero R Mor G Macrophages and apoptotic cell clearance during pregnancy. Am J Reprod Immunol. (2004) 514:275–82. 10.1111/j.1600-0897.2004.00156.x 15212680

[66] Nancy P Erlebacher A T cell behavior at the maternal-fetal interface. Int J Dev Biol. (2014) 582–4:189–98. 10.1387/ijdb.140054ae PMC4212519 25023685

[67] Gomez-Lopez N Vega-Sanchez R Castillo-Castrejon M Romero R Cubeiro-Arreola K Vadillo-Ortega F Evidence for a role for the adaptive immune response in human term parturition. Am J Reprod Immunol. (2013) 693:212–30. 10.1111/aji.12074 PMC3600361 23347265

[68] Huang Y Zhu XY Du MR Li DJ Human trophoblasts recruited T lymphocytes and monocytes into decidua by secretion of chemokine CXCL16 and interaction with CXCR6 in the first-trimester pregnancy. J Immunol. (2008) 1804:2367–75. 10.4049/jimmunol.180.4.2367 18250446

[69] Tsuda S Nakashima A Shima T Saito S. New paradigm in the role of regulatory T cells during pregnancy. Front Immunol. (2019) 10:573. 10.3389/fimmu.2019.00573 30972068 PMC6443934

[70] Wang SC Li YH Piao HL Hong XW Zhang D Xu YY. PD-1 and Tim-3 pathways are associated with regulatory CD8+ T-cell function in decidua and maintenance of normal pregnancy. Cell Death Dis. (2015) 6:e1738. 10.1038/cddis.2015.112 25950468 PMC4669692

[71] Tilburgs T Schonkeren D Eikmans M Nagtzaam NM Datema G Swings GM Human decidual tissue contains differentiated CD8+ effector-memory T cells with unique properties. J Immunol. (2010) 1857:4470–7. 10.4049/jimmunol.0903597 20817873

[72] Zhu J Yamane H Paul WE. Differentiation of effector CD4 T cell populations (^*^). Ann Rev Immunol. (2010) 28:445–89. 10.1146/annurev-immunol-030409-101212 20192806 PMC3502616

[73] Powell RM Lissauer D Tamblyn J Beggs A Cox P Moss P Decidual T cells exhibit a highly differentiated phenotype and demonstrate potential fetal specificity and a strong transcriptional response to IFN. J Immunol. (2017) 19910:3406–17. 10.4049/jimmunol.1700114 PMC5679367 28986438

[74] Hirahara K Nakayama T CD4+ T-cell subsets in inflammatory diseases: beyond the Th1/Th2 paradigm. Int Immunol. (2016) 284:163–71. 10.1093/intimm/dxw006 PMC4889886 26874355

[75] Lin Y Ren L Wang W Di J Zeng S Saito S Effect of TLR3 and TLR7 activation in uterine NK cells from non-obese diabetic (NOD) mice. J Reprod Immunol. (2009) 821:12–23. 10.1016/j.jri.2009.03.004 19560213

[76] Raghupathy R Makhseed M Azizieh F Hassan N Al-Azemi M Al-Shamali E Maternal Th1- and Th2-type reactivity to placental antigens in normal human pregnancy and unexplained recurrent spontaneous abortions. Cell Immunol. (1999) 1962:122–30. 10.1006/cimm.1999.1532 10527564

[77] Wu HX Jin LP Xu B Liang SS Li DJ Decidual stromal cells recruit Th17 cells into decidua to promote proliferation and invasion of human trophoblast cells by secreting IL-17. Cell Mol Immunol. (2014) 113:253–62. 10.1038/cmi.2013.67 PMC4085486 24633013

[78] Alijotas-Reig J Llurba E Gris JM Potentiating maternal immune tolerance in pregnancy: a new challenging role for regulatory T cells. Placenta. (2014) 354:241–8. 10.1016/j.placenta.2014.02.004 24581729

[79] La Rocca C Carbone F Longobardi S Matarese G The immunology of pregnancy: regulatory T cells control maternal immune tolerance toward the fetus. Immunol Lett. (2014) 1621 (Pt A):41–8. 10.1016/j.imlet.2014.06.013 24996040

[80] Samstein RM Josefowicz SZ Arvey A Treuting PM Rudensky AY Extrathymic generation of regulatory T cells in placental mammals mitigates maternal-fetal conflict. Cell. (2012) 1501:29–38. 10.1016/j.cell.2012.05.031 PMC3422629 22770213

[81] Campbell DJ Koch MA Phenotypical and functional specialization of FOXP3+ regulatory T cells. Nat Rev Immunol. (2011) 112:119–30. 10.1038/nri2916 PMC3289970 21267013

[82] Yamagiwa S Gray JD Hashimoto S Horwitz DA A role for TGF-beta in the generation and expansion of CD4+CD25+ regulatory T cells from human peripheral blood. J Immunol. (2001) 16612:7282–9. 10.4049/jimmunol.166.12.7282 11390478

[83] Zheng SG Gray JD Ohtsuka K Yamagiwa S Horwitz DA Generation *ex vivo* of TGF-β-producing regulatory T cells from CD4+CD25– precursors. J Immunol. (2002) 1698:4183–9. 10.4049/jimmunol.169.8.4183 12370347

[84] Wang WJ Liu FJ Qu HM Hao CF Qu QL Xiong W Regulation of the expression of Th17 cells and regulatory T cells by IL-27 in patients with unexplained early recurrent miscarriage. J Reprod Immunol. (2013) 991–2:39–45. 10.1016/j.jri.2013.04.002 23731956

[85] Thornton AM Korty PE Tran DQ Wohlfert EA Murray PE Belkaid Y Expression of Helios, an Ikaros transcription factor family member, differentiates thymic-derived from peripherally induced Foxp3+ T regulatory cells. J Immunol. (2010) 1847:3433–41. 10.4049/jimmunol.0904028 PMC3725574 20181882

[86] Elkord E Al-Ramadi BK Helios expression in FoxP3(+) T regulatory cells. Exp Opin Biol Ther. (2012) 1211:1423–5. 10.1517/14712598.2012.711310 22827571

[87] Lin X Chen M Liu Y Guo Z He X Brand D Advances in distinguishing natural from induced Foxp3(+) regulatory T cells. Int J Clin Exp Pathol. (2013) 62:116–23. PMC3544233 23329997

[88] Mjosberg J Berg G Jenmalm MC Ernerudh J FOXP3+ regulatory T cells and T helper 1, T helper 2, and T helper 17 cells in human early pregnancy decidua. Biol Reprod. (2010) 824:698–705. 10.1095/biolreprod.109.081208 20018909

[89] Tilburgs T Roelen DL van der Mast BJ de Groot-Swings GM Kleijburg C Scherjon SA Evidence for a selective migration of fetus-specific CD4+CD25bright regulatory T cells from the peripheral blood to the decidua in human pregnancy. J Immunol. (2008) 1808:5737–45. 10.4049/jimmunol.180.8.5737 18390759

[90] Somerset DA Zheng Y Kilby MD Sansom DM Drayson MT Normal human pregnancy is associated with an elevation in the immune suppressive CD25+ CD4+ regulatory T-cell subset. Immunology. (2004) 1121:38–43. 10.1111/j.1365-2567.2004.01869.x PMC1782465 15096182

[91] Sasaki Y Sakai M Miyazaki S Higuma S Shiozaki A Saito S Decidual and peripheral blood CD4+CD25+ regulatory T cells in early pregnancy subjects and spontaneous abortion cases. Mol Hum Reprod. (2004) 105:347–53. 10.1093/molehr/gah044 14997000

[92] Jin LP Chen QY Zhang T Guo PF Li DJ The CD4+CD25 bright regulatory T cells and CTLA-4 expression in peripheral and decidual lymphocytes are down-regulated in human miscarriage. Clin Immunol. (2009) 1333:402–10. 10.1016/j.clim.2009.08.009 19766059

[93] Bao SH Wang XP De Lin Q Wang WJ Yin GJ Qiu LH Decidual CD4+CD25+CD127dim/- regulatory T cells in patients with unexplained recurrent spontaneous miscarriage. Eur J Obstet Gynecol Reprod Biol. (2011) 1551:94–8. 10.1016/j.ejogrb.2010.11.007 21130556

[94] Chang RQ Li DJ Li MQ The role of indoleamine-2,3-dioxygenase in normal and pathological pregnancies. Am J Reprod Immunol. (2018) 794:e12786 10.1111/aji.12786 29154462

[95] Miwa N Hayakawa S Miyazaki S Myojo S Sasaki Y Sakai M. IDO expression on decidual and peripheral blood dendritic cells and monocytes/macrophages after treatment with CTLA-4 or interferon-gamma increase in normal pregnancy but decrease in spontaneous abortion. Mol Hum Reprod. (2005) 1112:865–70. 10.1093/molehr/gah246 16421220

[96] Zhu C Anderson AC Schubart A Xiong H Imitola J Khoury SJ The Tim-3 ligand galectin-9 negatively regulates T helper type 1 immunity. Nat Immunol. (2005) 612:1245–52. 10.1038/ni1271 16286920

[97] Saito S Sasaki Y Sakai M CD4(+)CD25high regulatory T cells in human pregnancy. J Reprod Immunol. (2005) 652:111–20. 10.1016/j.jri.2005.01.004 15811516

[98] Piccinni MP T-cell cytokines in pregnancy. Am J Reprod Immunol. (2002) 475:289–94. 10.1034/j.1600-0897.2002.01104.x 12148544

[99] Blois SM Kammerer U Alba Soto C Tometten MC Shaikly V Barrientos G Dendritic cells: key to fetal tolerance? Biol Reprod. (2007) 774:590–8. 10.1095/biolreprod.107.060632 17596562

[100] Tagliani E Dendritic cell function at the maternal–fetal interface. Expert Rev Clin Immunol. (2011) 75:593–602. 10.1586/eci.11.52 PMC3216484 21895472

[101] Gardner L Moffett A Dendritic cells in the human decidua. Biol Reprod. (2003) 694:1438–46. 10.1095/biolreprod.103.017574 12826583

[102] Miyazaki S Tsuda H Sakai M Hori S Sasaki Y Futatani T Predominance of Th2-promoting dendritic cells in early human pregnancy decidua. J Leukoc Biol. (2003) 744:514–22. 10.1189/jlb.1102566 12960246

[103] Rieger L Honig A Sutterlin M Kapp M Dietl J Ruck P Antigen-presenting cells in human endometrium during the menstrual cycle compared to early pregnancy. J Soc Gynecol Investig. (2004) 117:488–93. 10.1016/j.jsgi.2004.05.007 15458747

[104] Kammerer U Eggert AO Kapp M McLellan AD Geijtenbeek TB Dietl J Unique appearance of proliferating antigen-presenting cells expressing DC-SIGN (CD209) in the decidua of early human pregnancy. Am J Pathol. (2003) 1623:887–96. 10.1016/S0002-9440(10)63884-9 PMC1868095 12598322

[105] Sheng YR Hu WT Wei CY Tang LL Liu YK Liu YY IL-33/ST2 axis affects the polarization and efferocytosis of decidual macrophages in early pregnancy. Am J Reprod Immunol. (2018) 796:e12836 10.1111/aji.12836 29500844

[106] Kammerer U Kruse A Barrientos G Arck PC Blois SM Role of dendritic cells in the regulation of maternal immune responses to the fetus during mammalian gestation. Immunol Investig. (2008) 375:499–533. 10.1080/08820130802191334 18716936

[107] Blois SM Barrientos G Garcia MG Orsal AS Tometten M Cordo-Russo RI Interaction between dendritic cells and natural killer cells during pregnancy in mice. J Mol Med. (2008) 867:837–52. 10.1007/s00109-008-0342-2 18506412

[108] Leno-Duran E Munoz-Fernandez R Olivares EG Tirado-Gonzalez I Liaison between natural killer cells and dendritic cells in human gestation. Cell Mol Immunol. (2014) 115:449–55. 10.1038/cmi.2014.36 PMC4197206 24954224

[109] Takenaka MC Quintana FJ Tolerogenic dendritic cells. Sem Immunopathol. (2017) 392:113–20. 10.1007/s00281-016-0587-8 PMC5296314 27646959

[110] Dzionek A Fuchs A Schmidt P Cremer S Zysk M Miltenyi S BDCA-2, BDCA-3, and BDCA-4: three markers for distinct subsets of dendritic cells in human peripheral blood. J Immunol. (2000) 16511:6037–46. 10.4049/jimmunol.165.11.6037 11086035

[111] Hunt JS. Stranger in a strange land. Immunol Rev. (2006) 213:36–47. 10.1111/j.1600-065X.2006.00436.x 16972895 PMC1637092

[112] Hackmon R Pinnaduwage L Zhang J Lye SJ Geraghty DE Dunk CE. Definitive class I human leukocyte antigen expression in gestational placentation: HLA-F, HLA-E, HLA-C, and HLA-G in extravillous trophoblast invasion on placentation, pregnancy, and parturition. Am J Reprod Immunol. (2017) 77. 10.1111/aji.12643 28185362

[113] Ferreira LMR Meissner TB Tilburgs T Strominger JL HLA-G: at the interface of maternal-fetal tolerance. Trends Immunol. (2017) 384:272–86. 10.1016/j.it.2017.01.009 28279591

[114] Long EO. Regulation of immune responses through inhibitory receptors. Ann Rev Immunol. (1999) 17:875–904. 10.1146/annurev.immunol.17.1.875 10358776

[115] Shakhawat A Shaikly V Elzatma E Mavrakos E Jabeen A Fernandez N Interaction between HLA-G and monocyte/macrophages in human pregnancy. J Reprod Immunol. (2010) 851:40–6. 10.1016/j.jri.2010.02.004 20356631

[116] Gregori S Amodio G Quattrone F Panina-Bordignon P. HLA-G orchestrates the early interaction of human trophoblasts with the maternal niche. Front Immunol. (2015) 6:128. 10.3389/fimmu.2015.00128 25870595 PMC4378286

[117] Petroff MG Chen L Phillips TA Azzola D Sedlmayr P Hunt JS B7 family molecules are favorably positioned at the human maternal-fetal interface. Biol Reprod. (2003) 685:1496–504. 10.1095/biolreprod.102.010058 12606489

[118] Zhou J Xiao X-M Wu Y-H Expression of interferon-γ in decidual natural killer cells from women with hypertensive disorder complicating pregnancy. J Obstet Gynaecol Res. (2014) 403:670–6. 10.1111/jog.12216 24246020

[119] Cartwright JE James-Allan L Buckley RJ Wallace AE. The role of decidual NK cells in pregnancies with impaired vascular remodelling. J Reprod Immunol. (2017) 119:81–4. 10.1016/j.jri.2016.09.002 27680579

[120] Williams PJ Bulmer JN Searle RF Innes BA Robson SC Altered decidual leucocyte populations in the placental bed in pre-eclampsia and foetal growth restriction: a comparison with late normal pregnancy. Reproduction. (2009) 1381:177–84. 10.1530/REP-09-0007 19357130

[121] Eide IP Rolfseng T Isaksen CV Mecsei R Roald B Lydersen S Serious foetal growth restriction is associated with reduced proportions of natural killer cells in decidua basalis. Virchows Arch Int J Pathol. (2006) 4483:269–76. 10.1007/s00428-005-0107-z 16328353

[122] Rieger L Segerer S Bernar T Kapp M Majic M Morr AK. Specific subsets of immune cells in human decidua differ between normal pregnancy and preeclampsia–a prospective observational study. Reprod Biol Endocrinol. (2009) 7:132. 10.1186/1477-7827-7-132 19930648 PMC2789084

[123] Milosevic-Stevanovic J Krstic M Radovic-Janosevic D Popovic J Tasic M Stojnev S Number of decidual natural killer cells & macrophages in pre-eclampsia. Indian J Med Res. (2016) 1446:823–30. 10.4103/ijmr.IJMR_776_15 PMC5433275 28474619

[124] Stallmach T Hebisch G Orban P Lu X Aberrant positioning of trophoblast and lymphocytes in the feto-maternal interface with pre-eclampsia. Virchows Arch Int J Pathol. (1999) 4343:207–11. 10.1007/s004280050329 10190299

[125] Schonkeren D van der Hoorn ML Khedoe P Swings G van Beelen E Claas F Differential distribution and phenotype of decidual macrophages in preeclamptic versus control pregnancies. Am J Pathol. (2011) 1782:709–17. 10.1016/j.ajpath.2010.10.011 PMC3069820 21281803

[126] Tsao FY Wu MY Chang YL Wu CT Ho HN M1 macrophages decrease in the deciduae from normal pregnancies but not from spontaneous abortions or unexplained recurrent spontaneous abortions. J Formos Med Assoc. (2018) 1173:204–11. 10.1016/j.jfma.2017.03.011 28465068

[127] Reister F Frank HG Heyl W Kosanke G Huppertz B Schröder W The distribution of macrophages in spiral arteries of the placental bed in pre-eclampsia differs from that in healthy patients. Placenta. (1999) 202:229–33. 10.1053/plac.1998.0373 10195746

[128] Milosevic-Stevanovic J Krstic M Stefanovic M Zivadinovic R Vukomanovic P Trajkovic-Dinic SP T lymphocytes in the third trimester decidua in preeclampsia. Hypertens Pregnancy. (2019) 381:52–7. 10.1080/10641955.2019.1575393 30744453

[129] Rahimzadeh M Norouzian M Arabpour F Naderi N Regulatory T-cells and preeclampsia: an overview of literature. Exp Rev Clin Immunol. (2016) 122:209–27. 10.1586/1744666X.2016.1105740 26580672

[130] Osborne LM Brar A Klein SL. The role of Th17 cells in the pathophysiology of pregnancy and perinatal mood and anxiety disorders. Brain Behav Immun. (2019) 76:7–16. 10.1016/j.bbi.2018.11.015 30465878 PMC6359933

[131] Scholz C Toth B Santoso L Kuhn C Franz M Mayr D Distribution and maturity of dendritic cells in diseases of insufficient placentation. Am J Reprod Immunol. (2008) 603:238–45. 10.1111/j.1600-0897.2008.00619.x 18782285

[132] Askelund K Liddell HS Zanderigo AM Fernando NS Khong TY Stone PR CD83(+)dendritic cells in the decidua of women with recurrent miscarriage and normal pregnancy. Placenta. (2004) 252–3:140–5. 10.1016/S0143-4004(03)00182-6 14972446

[133] Wang J Su L Zhu T Shen M [Changes in the subsets of dendritic cells and T cells in peripheral blood of patients with preeclampsia]. Chin J Cell Mol Immunol. (2013) 291:72–5. 23294721

[134] Zhang J Dunk CE Shynlova O Caniggia I Lye SJ. TGFb1 suppresses the activation of distinct dNK subpopulations in preeclampsia. EBioMedicine. (2019) 39:531–9. 10.1016/j.ebiom.2018.12.015 30579870 PMC6355656

[135] Renaud SJ Postovit LM Macdonald-Goodfellow SK McDonald GT Caldwell JD Graham CH Activated macrophages inhibit human cytotrophoblast invasiveness *in vitro*. Biol Reprod. (2005) 732:237–43. 10.1095/biolreprod.104.038000 15800179

[136] Huang SJ Chen CP Schatz F Rahman M Abrahams VM Lockwood CJ Pre-eclampsia is associated with dendritic cell recruitment into the uterine decidua. J Pathol. (2008) 2143:328–36. 10.1002/path.2257 18069648

[137] Fraser R Whitley GS Johnstone AP Host AJ Sebire NJ Thilaganathan B Impaired decidual natural killer cell regulation of vascular remodelling in early human pregnancies with high uterine artery resistance. J Pathol. (2012) 2283:322–32. 10.1002/path.4057 PMC3499663 22653829

[138] King K Smith S Chapman M Sacks G Detailed analysis of peripheral blood natural killer (NK) cells in women with recurrent miscarriage. Hum Reprod. (2010) 251:52–8. 10.1093/humrep/dep349 19819893

[139] Seshadri S Sunkara SK Natural killer cells in female infertility and recurrent miscarriage: a systematic review and meta-analysis. Hum Reprod Update. (2014) 203:429–38. 10.1093/humupd/dmt056 24285824

[140] Lachapelle MH Miron P Hemmings R Roy DC Endometrial T, B, and NK cells in patients with recurrent spontaneous abortion. Altered profile and pregnancy outcome. J Immunol. (1996) 15610:4027–34. 8621945

[141] Ratsep MT Felker AM Kay VR Tolusso L Hofmann AP Croy BA Uterine natural killer cells: supervisors of vasculature construction in early decidua basalis. Reproduction. (2015) 1492:R91–102. 10.1530/REP-14-0271 25342175

[142] Hutter S Heublein S Knabl J Andergassen U Vrekoussis T Makrigiannakis A Macrophages: are they involved in endometriosis, abortion and preeclampsia and how? J Nippon Med Sch. (2013) 802:97–103. 10.1272/jnms.80.97 23657062

[143] Guenther S Vrekoussis T Heublein S Bayer B Anz D Knabl J Decidual macrophages are significantly increased in spontaneous miscarriages and over-express FasL: a potential role for macrophages in trophoblast apoptosis. Int J Mol Sci. (2012) 137:9069–80. 10.3390/ijms13079069 PMC3430283 22942752

[144] Wang WJ Hao CF Lin QD Dysregulation of macrophage activation by decidual regulatory T cells in unexplained recurrent miscarriage patients. J Reprod Immunol. (2011) 921–2:97–102. 10.1016/j.jri.2011.08.004 22015003

[145] Yamada H Takeda M Maezawa Y Ebina Y Hazama R Tanimura K A high dose intravenous immunoglobulin therapy for women with four or more recurrent spontaneous abortions. ISRN Obstet Gynecol. (2012) 2012:512732 10.5402/2012/512732 22997588 PMC3446652

[146] Wang SW Zhong SY Lou LJ Hu ZF Sun HY Zhu HY The effect of intravenous immunoglobulin passive immunotherapy on unexplained recurrent spontaneous abortion: a meta-analysis. Reprod Biomed Online. (2016) 336:720–36. 10.1016/j.rbmo.2016.08.025 27720163

[147] Jianjun Z Yali H Zhiqun W Mingming Z Xia Z Imbalance of T-cell transcription factors contributes to the Th1 type immunity predominant in pre-eclampsia. Am J Reprod Immunol. (2010) 631:38–45. 10.1111/j.1600-0897.2009.00763.x 19912158

[148] Cerdeira AS Kopcow HD Karumanchi SA Regulatory T cells in preeclampsia: some answers, more questions? Am J Pathol. (2012) 1816:1900–2. 10.1016/j.ajpath.2012.09.020 PMC3509764 23063658

[149] Qian ZD Huang LL Zhu XM. An immunohistochemical study of CD83- and CD1a-positive dendritic cells in the decidua of women with recurrent spontaneous abortion. Eur J Med Res. (2015) 20:2. 10.1186/s40001-014-0076-2 25563385 PMC4301856

[150] Tirado-Gonzalez I Munoz-Fernandez R Blanco O Leno-Duran E Abadia-Molina AC Olivares EG Reduced proportion of decidual DC-SIGN+ cells in human spontaneous abortion. Placenta. (2010) 3111:1019–22. 10.1016/j.placenta.2010.09.008 20934749

[151] Santner-Nanan B Peek MJ Khanam R Richarts L Zhu E St. Groth B Systemic increase in the ratio between Foxp3+ and IL-17-producing CD4+ T cells in healthy pregnancy but not in preeclampsia. J Immunol. (2009) 183:7023–30. 10.4049/jimmunol.0901154 19915051

